# Postoperative hemorrhage after biomedical glue sling technique in microvascular decompression for vertebrobasilar artery-associated cranial nerve diseases: A retrospective study of 14 cases

**DOI:** 10.3389/fsurg.2022.943848

**Published:** 2023-01-06

**Authors:** Jiang Liu, Yuxiao Shen, Kelisitan Xiayizhati, Yanbing Yu

**Affiliations:** ^1^ Department of Neurosurgery, China-Japan Friendship Hospital, Beijing, China; ^2^ Department of Neurosurgery, Peking University China-Japan Friendship School of Clinical Medicine, Beijing, China; ^3^ Department of Neurosurgery, People’s Hospital of Xinjiang Uygur Autonomous Region, Urumqi, China

**Keywords:** microvascular decompressionage, postoperative hemorrhage, biomedical glue sling technique, vertebrobasliar artery, cranial nerve neuropathy

## Abstract

**Background:**

The biomedical glue sling technique is a convenient and effective method for vertebrobasilar artery-associated cranial nerve diseases but postoperative hemorrhage is poorly understood.

**Methods:**

We retrospectively reviewed 14 of 1157 patients associated with cranial nerve diseases who were subjected to the biomedical glue sling technique in microvascular decompression at our hospital from January 2015 to January 2020.

**Results:**

There were 14 patients with cranial nerve diseases included in this study. A clinical diagnosis of postoperative hemorrhage was made after an average of 41.75 h (ranging between 0.5 and 95 h). A cerebellopontine angle hemorrhage was presented in 5 patients, while basal ganglia hemorrhage was observed in 2 patients. Both a cerebellopontine angle and brainstem hemorrhage was seen in 1 patient. Distal supratentorial subdural hemorrhage was recorded in 6 patients. The correlation coefficient was −0.1601 (*p* = 0.7094) between the standard deviation of systolic blood pressure and the Hemphill Score, −0.2422 (*p* = 0.5633) between the coefficient of variation of systolic blood pressure and the Hemphill Score, and −0.0272 (*p* = 0.9489) between the range of systolic blood pressure and the Hemphill Score.

**Conclusions:**

The incidence of postoperative hemorrhage after MVD with the biomedical glue sling technique is higher than with traditional MVD and most cases have a favorable prognosis. Postoperative symptoms are the main area of concern and changes in symptoms usually suggest the occurrence of hemorrhage. Several factors, including surgical procedures, the release of CSF, and blood pressure might be associated with hemorrhaging. We still believe such a technique is an efficient approach to treating complicated cranial nerve diseases.

## Introduction

Hemifacial spasms (HFS) and trigeminal neuralgia (TN) are the most common cranial nerve diseases (CND) and are generally caused by vascular-nerve conflicts. At present, microvascular decompression (MVD) is the most effective treatment for CND. However, vertebrobasilar artery (VBA)-associated CND bring some challenges, a relatively lower curative ratio, and higher postoperative complication morbidity. Approximately 0.7%–17.5% of HFS cases and 0.9%–7.7% of TN cases are associated with VBA (1–4), while some articles reported that a recurrence rate of MVD for VBA-associated CND was relatively high (5–8).

To achieve better outcomes, surgeons tried several techniques wherein the biomedical glue sling technique was a relatively convenient method to deal with VBA (9). The effectiveness of the biomedical glue sling technique for VBA-associated CNDs has been demonstrated in some reports (9, 10), but a few studies have focused on postoperative hemorrhages. In our research, we reviewed a series of cases of postoperative hemorrhage after microvascular decompression with the biomedical glue sling technique was used and we analyzed reasons to reduce the incidence of postoperative hemorrhage.

## Materials and methods

### Patient population

Between January 2015 and January 2020, 1,157 patients presenting a CND underwent MVD with the biomedical glue sling technique at our hospital, wherein 14 patients developed postoperative hemorrhage. We treated 7 men and 7 women, with a mean age of 55.9 years (ranging from 25 to 76). The duration of symptoms ranged from 4 months to 20 years. HFS was diagnosed in 9 patients while 4 patients were diagnosed with TN. The coexistence of HFS and TN occurred in 1 patient. The left side was affected in 9 patients, and the right side in 5 patients. Of the 14 patients, 6 were diagnosed with preoperative hypertension (Table 1). The diagnoses of HFS and TN were based on typical clinical symptoms. Preoperative MRIs or CTs were carried out to exclude other conditions and assess the state of the brain parenchyma and vessels. The study protocol was reviewed and approved by the Ethics Committee (IEC) of our hospital. Because this is a retrospective study with no potential conflicts of interest and no potential damage, the need for informed consent was waived.

**Table 1:** Summary of 14 cases with postoperative hemorrhage.

Cases	Age (years)/Sex	CND	Side	Duration (years)	Underlying Diseases	Offending Vessels	Outcome(Kondon/BNI)^a^	Hemorrhage Site	Time^b^ (hours)
1	67/F	HFS	L	3	/	AICA + VA	E0	CPA	20
2	54/M	HFS	L	2	Hypertension	AICA + PICA + VA	E0	CPA	27
3	56/M	TN	L	10	/	SCA + BA	BNI I	basal ganglia	42
4	64/F	HFS	R	20	Hypertension	PICA + BA	E1	CPA	56
5	76/F	HFS	L	1	Hypertension	AICA + VA	E0	dSSDH	69
6	50/F	TN	L	1	/	BA	BNI I	CPA	94
7	42/F	TN	R	10	/	SCA + BA	/	dSSDH	0.5
8	61/M	HFS	L	10	Hypertension	AICA + BA	E0	dSSDH	16
9	61/F	HFS	R	10	Hypertension	AICA + PICA + VA	E0	dSSDH	95
10	67/M	HFS & TN	L	12 (HFS) 2 months (TN)	/	AICA SCA + BA	E1 & BNI I	CPA & fourth ventricle	45
11	63/M	HFS	L	10	/	PICA + VA	E0	dSSDH	66
12	35/M	HFS	L	4 months	/	AICA + VA	E0	CPA & brainstem	26
13	61/F	TN	R	10	Hypertension	SCA + VA	/ (death)	basal ganglia	3
14	25/M	HFS	R	4 months	/	PICA + VA	E0	dSSDH	72

AICA, anterior inferior cerebellar artery; VA, vertebral artery; PICA, posterior inferior cerebellar artery; SCA, superior cerebellar artery; BA, basilar artery; CPA, cerebellopontine angle; dSSDH, distal supratentorial subdural hemorrhage, /= NA.

^a^ Immediate postoperative outcome was evaluated, and 2 patients could not be assessed because of immediate postoperative hemorrhage.

^b^ Time for the onset of postoperative hemorrhage to be diagnosed clinically.

### Surgical procedures

A standardized lateral suboccipital retrosigmoid approach was performed on all patients. Patients were placed in the lateral decubitus position with the head rotated approximately 10° away from the affected side and the vertex dropped 15° towards the floor. A 2 × 2 cm bone window was opened at the dorsal side of the sigmoid sinus. After a sharp dissection of the arachnoid membrane, while slowly releasing the cerebrospinal fluid, the lower cranial nerves were distinctly exposed, delicately examining the anatomical relationships between cranial nerves and vessels in the cerebellopontine angle. Teflon pads were first inserted as stents between the brainstem and the VBA. The VBA was pushed smoothly toward the petrosal apex. After the VBA was shifted to its appropriate position, a piece of gelfoam with biomedical glue was used to attach the VBA to the petrosal apex. At that point, there was enough space to perform the surgical procedure. Other vessels were fully decompressed in sequence. Finally, the incision was closed with sutures in a watertight pattern, without the placement of drainage.

### Intraoperative monitoring and clinical evaluation

During the operation, we measured the blood pressure every 5 min. A brainstem auditory evoked potential (BAEP) was used to monitor the hearing audial function of patients. We calculated the standard deviation (SD), coefficient of variation (CV), and range of systolic blood pressure (SBP) in CND patients with intracerebral hemorrhage (ICH) or cerebellopontine angle (CPA) hemorrhage. The efficacy of such an operation was assessed using Barrow Neurological Institute (BNI) pain intensity score in TN patients, while another scoring system reported by Kondon et al. was used in HFS patients (Table 2) (11, 12). The Hemphill Score was used for evaluating the severity of the hemorrhage, and a postoperative CT was carried out to assess the progress of the hemorrhage itself. A regular CT scan was used to evaluate all patients on the fourth day after surgery at our facility. We calculated the correlation of the listed values using logistic regression analysis. Statistical analysis was conducted using STATA, version 15.

**Table 2:** Evaluation of HFS and TN.

HFS	
Grade	Definition
E0	Complete disappearance of spasm
E1	Occasional slight spasm
E2	Moderate spasms, apparently persisting
E3	Not cured
TN	
Grade	
BNII	No trigeminal pain, no medication
BNIII	Occasional pain, not requiring medication
BNIIII	Some pain, adequately controlled with medication
BNIIV	Some pain, not adequately controlled with medication
BNIV	Severe pain/no pain relief

## Results

**Figure 1:**
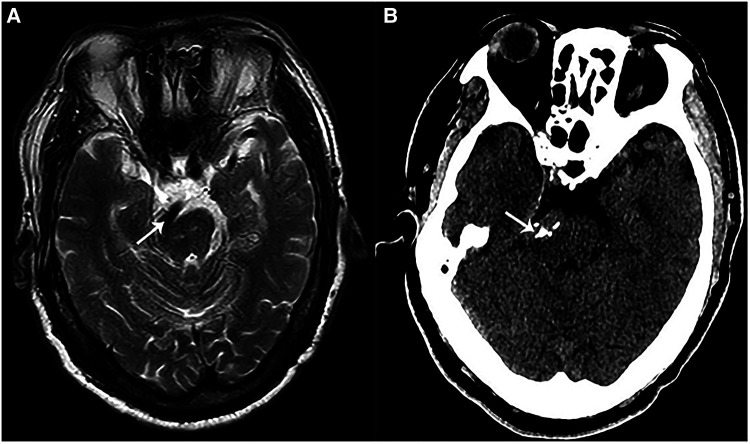
Preoperative imaging of a patient with VBA-associated HFS. (**A**) A preoperative magnetic resonance imaging (MRI) showed that VBA (white arrow) deformed and compressed the brainstem at pons. (**B**) A preoperative CT showed calcified VBA (white arrow) which compressed the brainstem at pons.

**Figure 2:**
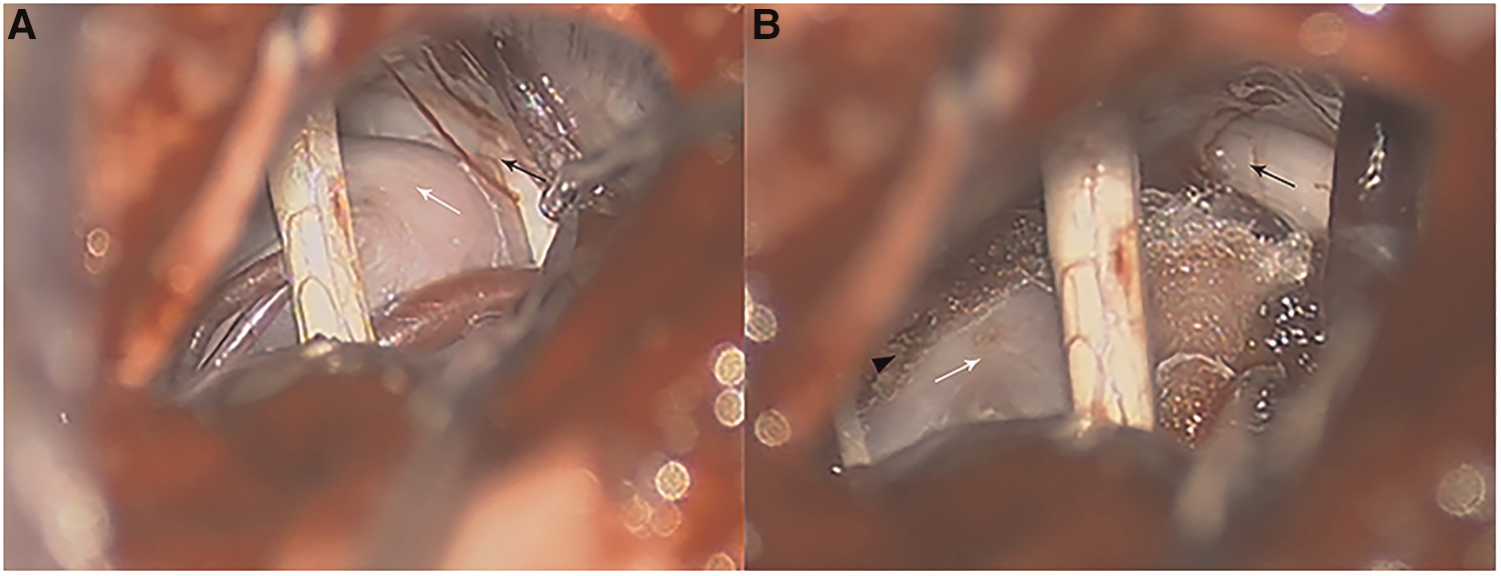
Intraoperative photographs in a patient with VBA-associated TN. (**A**) Intraoperative photograph of the anatomy relationship of a calcified BA (white arrow) and the trigeminal nerve (black arrow). (**B**) Teflon pads were inserted between the trigeminal nerve (black arrow) and BA (white arrow) which were fixed on the petrosal apex through a small piece of dry gelfoam with biomedical glue (black arrowhead) and transposed from the trigeminal nerve (black arrow).

The VBA was observed to be the offending vessel in all 14 patients, wherein the calcification of the VBA was found in 4 patients through a preoperative CT scan (Figures 1, 2). Besides, other vessels were also involved in these cases, including the anterior inferior cerebellar artery (AICA) in 7 cases, the posterior inferior cerebellar artery (PICA) in 5 cases, and the superior cerebellar artery (SCA) in 5 cases.

Of those patients with HFS only, 8 out of 9 (88.9%) experienced complete spasm relief (E0) immediately after surgery, and 1 patient experienced partial relief (E1). The symptoms in 2 patients with TN only disappeared (BNI I) after surgery, and 2 more patients could not be evaluated because of immediate postoperative hemorrhage. The patient with concurrent HFS and TN experienced complete relief of pain (BNI I) but the spasm still occurred (E1). During the follow-up, spasms in 2 patients (E1) gradually disappeared.

**Figure 3:**
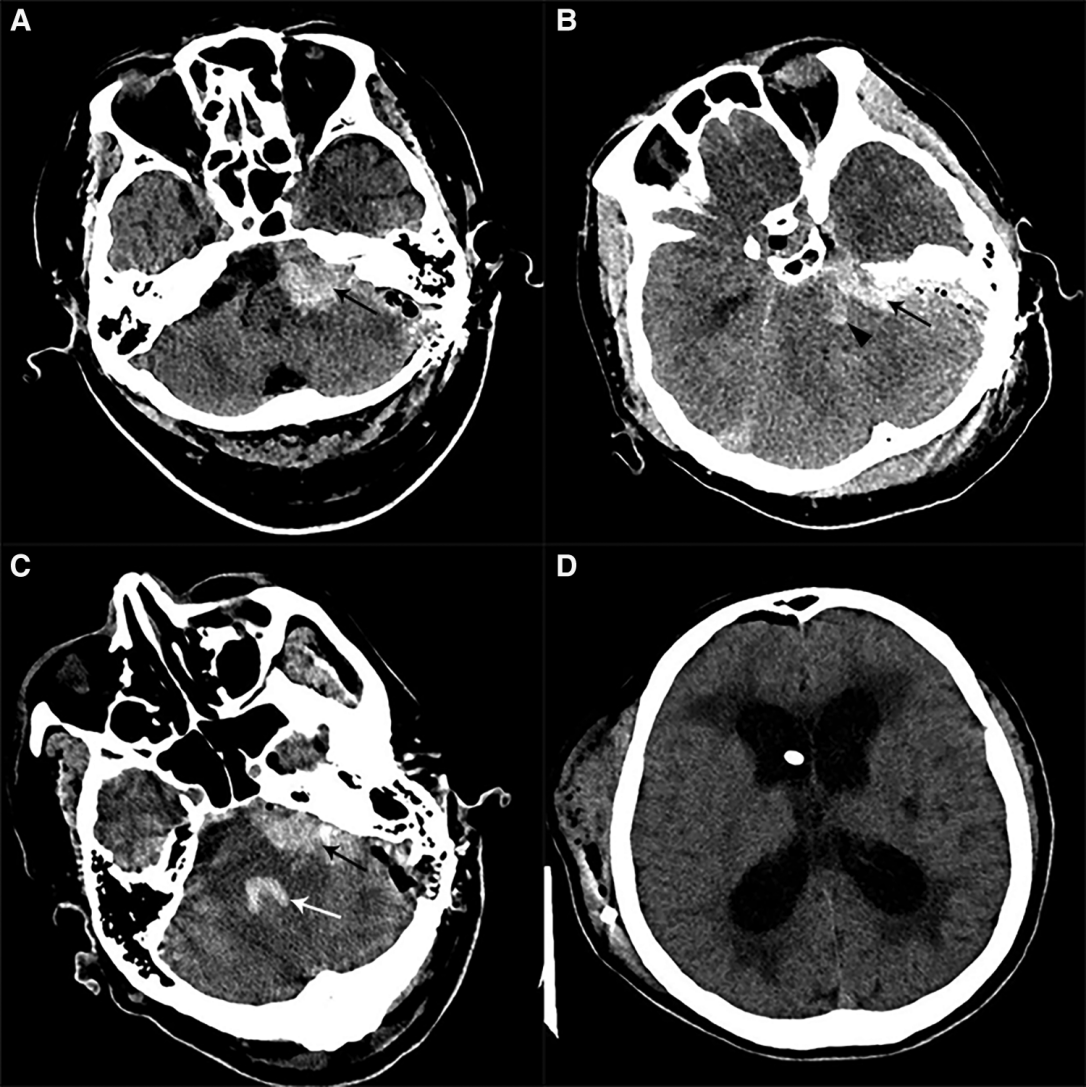
Postoperative imaging of patients with CPA hemorrhage. (**A**) Postoperative CT in a patient with left HFS showed a left CPA hemorrhage alone (black arrow). (**B**) Postoperative CT in one patient with left HFS showed CPA hemorrhage (black arrow) combined with brainstem hemorrhage (black arrowhead). (**C**) Postoperative CT in a patient with left HFS and TN showed a left CPA hemorrhage (black arrow) involving the fourth ventricle (white arrow). (**D**) We performed a V-P shunt on the patient who had experienced hydrocephalus two months after the CPA hemorrhage. CT showed a lateral ventricle enlargement, periventricular hydrocephalus, and distal stoma of the duct.

**Figure 4:**
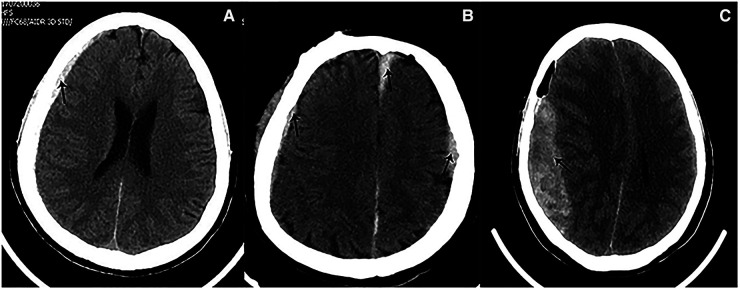
Postoperative imaging of patients with SDH. (**A**) Postoperative CT in a patient with left HFS showed a right frontal SDH (black arrow). (**B**) Postoperative CT in a patient with left HFS showed multiple sites of SDH (black arrow), involving bilateral temporal lobes and the cerebral falx. (**C**) Postoperative CT on a patient with right TN showed that a massive right temporal SDH (black arrow) lead to the displacement of the midline.

**Figure 5:**
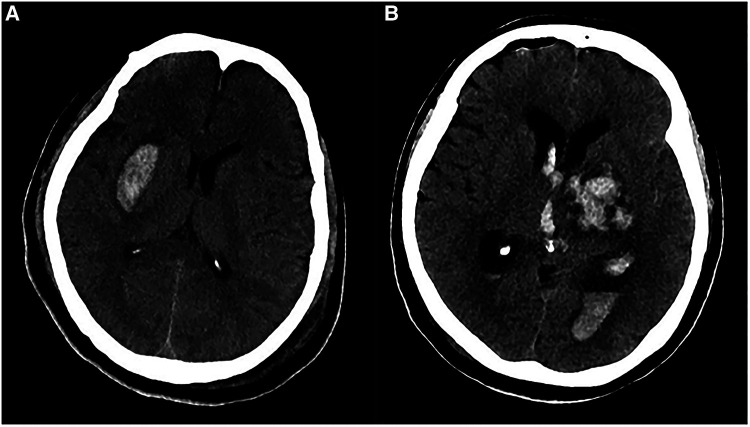
Postoperative imaging of patients with basal ganglia hemorrhage. (**A**) Postoperative CT in a patient with left TN showed a right external capsule hemorrhage. (**B**) Postoperative CT in a patient with right TN showed a left basal ganglia hemorrhage which ruptured to ventricles.

The average time for the clinical diagnosis of the onset of postoperative hemorrhage was 41.75 h (ranging between 0.5 h and 95 h). Consciousness disturbance occurred in 7 patients, severe headache in 11 patients, a decrease in muscle strength in 5 patients, and hemiplegia in 3 patients (Table 3). CPA hemorrhage presented in 5 patients and 2 patients developed basal ganglia hemorrhage. Both CPA and brainstem hemorrhage was observed in 1 patient. Distant supratentorial subdural hemorrhage (SDH) occurred in 6 patients (Figures 3–5). Out of 14 patients, 13 gradually recovered thanks to systemic treatments, and hydrocephalus occurred 2 months after hemorrhage in 1 patient who was subjected to a ventriculoperitoneal shunt, although 1 patient with basal ganglia hemorrhage died because of severe pulmonary infection while in the ICU (Table 1).

**Table 3:** Symptoms of patients with postoperative hemorrhage.

Symptoms	Cases
Mild headache	3
Severe headache	11
Consciousness disturbance	7
Decrease in muscle strength	5
Hemiplegia	3

The SD, CV, range of SBP, and the Hemphill Score of patients with CPA hemorrhage and ICH were presented in Table 4. The correlation coefficient was −0.1601 (*p* = 0.7094) between SD of SBP and the Hemphill Score, −0.2422 (*p* = 0.5633) between the CV of SBP and the Hemphill Score, −0.0272 (*p* = 0.9489) between the range of SBP and the Hemphill Score.

**Table 4:** Results of statistical analysis involving postoperative ICH patients.

Cases	SD of SBP	CV of SBP	Range of SBP	Hemphill Score
1	11.11	0.105	44	1
2	6.250	0.685	21	1
3	14.94	0.160	42	1
4	9.76	0.101	31	2
6	7.14	0.061	23	1
10	17.11	0.141	60	2
12	6.05	0.058	26	3
13	7.46	0.076	32	2
**Correlation coefficient with Hemphill Score**				
**SD**	**CV**	**Range**		** **
**−**0.1601 (*p* = 0.7094)	−0.2422 (*p* = 0.5633)	−0.0272 (*p* = 0.9489)		

SD, standard deviation; CV, coefficient of variation; SBP, systolic blood pressure.

## Discussion

The effectiveness and safety of MVD for the treatment of TN and HFS have been demonstrated, but difficult decompression during surgery significantly increases the risks. VBA-associated CND is a difficult type of compression in MVD. There is no standard method for the management of offending vessels involving VBA. Ting Lei et al. believed that traditional MVD was effective for TN caused by VBA (1). Vicente Vanaclocha et al. regarded the technique of inducing a dural scar to repair the BA in its new position, away from the trigeminal nerve, as simple, not technically demanding, and highly effective (3). Seong Ho Lee et al. deemed that the transposition of the VBA using a bioglue-coated Teflon sling and fixing other offending vessels to the transposed VBA with applications of biomedical glue was a safe and effective surgical technique for HFS involving VBA (13), while Naoki Otani et al. used human fibrinogen and fibrin glue to suspend the offending vessels. Among the 27 patients included in this study, only one presented with hearing loss (14). In our previous study, we used the biomedical glue sling technique to treat TN patients. Out of 22 involved patients, 20 experienced complete relief from pain (BNI I) after surgery, and only 1 patient complained of postoperative hypoacusis, which resolved within 2 months (9). It can be seen that there is currently no standard VBA suspension technique, and prognosis and postoperative complications of suspension surgery are still poorly understood compared to traditional MVD. In our study, we reviewed the clinical materials of 14 patients with postoperative hemorrhage, a relatively severe complication, to examine the reasons for its occurrence.

The most severe postoperative complication from traditional MVD is hemorrhage, the incidence of which is about 0.12%–2% (15). In our study, the incidence of postoperative hemorrhage associated with the biomedical glue sling technique in MVD is about 1.2%, which is marginally higher than that of traditional MVD performed in recent years. Thus, the diagnosis of postoperative hemorrhage at an early stage is critical. In our study, consciousness disturbance occurred in 7 patients, severe headache in 11 patients, a decrease in muscle strength in 5 patients, and hemiplegia in 3 patients. These symptoms suggested the possibility of postoperative hemorrhage and a CT scan should be carried out immediately. In addition, some patients who complained of a mild headache were diagnosed with postoperative hemorrhage through regular postoperative CT scans. Those patients usually developed SDH and had a favorable prognosis. For patients with CPA hemorrhage or ICH, we performed surgery, including decompressive craniectomy and hematoma evacuation, along with ventricle external puncture drainage. For patients with hydrocephalus caused by postoperative hemorrhage, a ventricular-peritoneal shunt represented an alternative approach instead of conservative therapy, because the circulation of cerebrospinal fluid (CSF) was difficult to establish on its own.

CPA was a common site for postoperative hemorrhage in MVD. As for patients with VBA-associated CNDs, vessels were usually in a poor condition. In our study, we found VBA atherosclerosis in 4 patients through CT scans and surgery. Moreover, the majority of patients with VBA atherosclerosis had a history of long-term use of oral antiplatelet drugs, which may have caused coagulation disorders. These factors could have contributed to postoperative hemorrhage in these patients. Therefore, the mentioned patients had a higher risk of bleeding than non-VBA patients, and intraoperative procedures further increased the probability of hemorrhage. First, a sharp dissection of the arachnoid membrane can cause vasoconstriction of injured small vessels in the arachnoid membrane, and these small vessels could end in staxis after the operation. The use of biomedical glue in suspension also has a direct thermal burning effect, which under certain circumstances may result in direct or indirect harm to related vessels. In addition, the VBA sling technique could inevitably lead to the displacement of some perforating branches. Said perforating branches continued to be in a high-tension state after being suspended. Once the vessel spasm of the perforating branches was relieved, some bleeding occurred. This demonstrated that the degree of suspended VBA must be based on its mobility, and excessive traction could result in a higher risk of postoperative hemorrhage. In some specific cases, we abandoned the biomedical glue sling technique to avoid postoperative hemorrhage, based on multiple considerations. Displacement of associated vessels, including small branches of VBA and perforating vessels supplying the brainstem, was the most plausible reason for postoperative hemorrhage in MVD with the biomedical glue sling technique.

**Figure 6:**
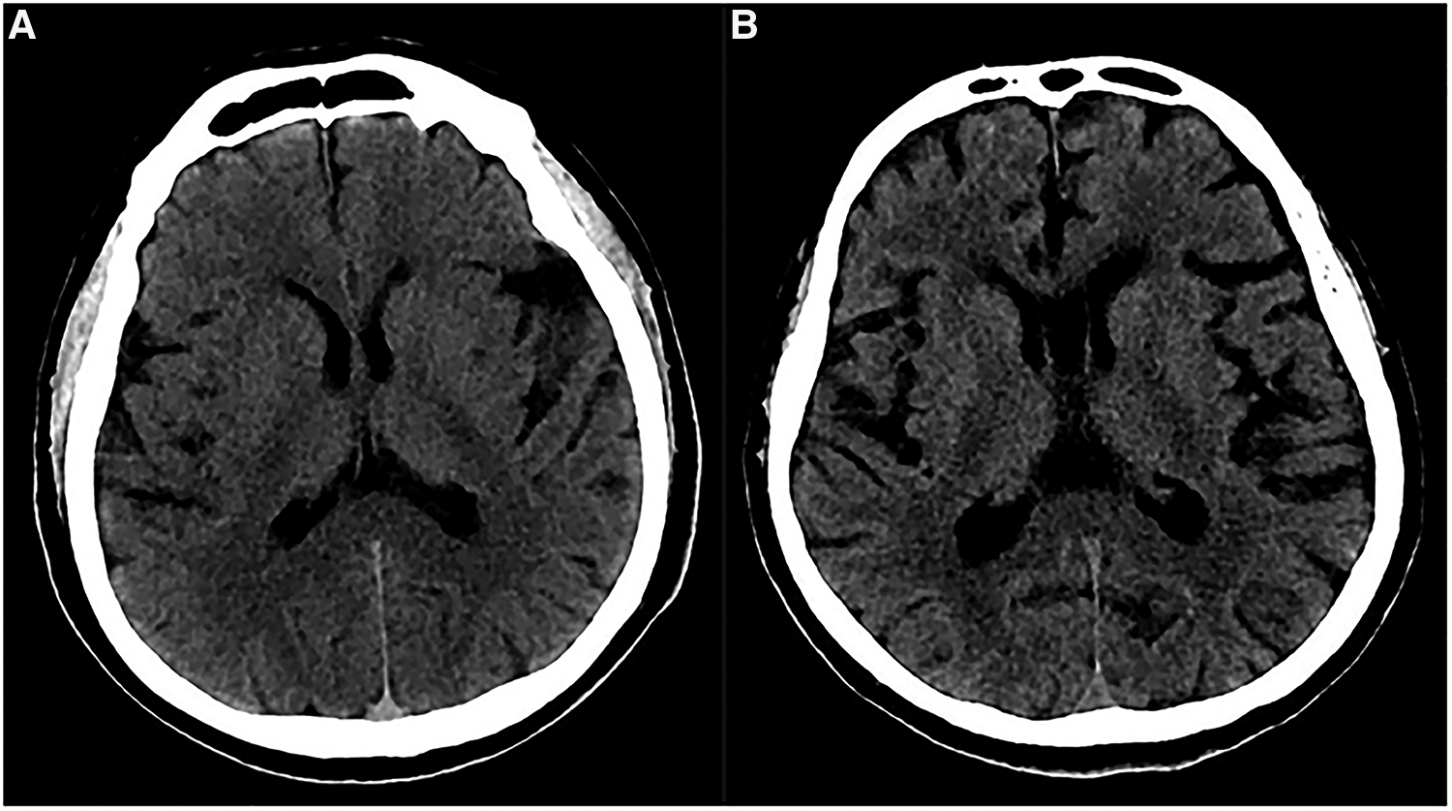
Preoperative imaging of patients with cerebral atrophy. (**A**) Preoperative CT showed a slight widened Sylvian fissure. (**B**) Preoperative CT showed more obvious widened cerebral sulcus and fissures as compared to A.

In our study, 6 patients (42.9%) developed distal supratentorial SDH, which is the most common postoperative hemorrhage for these cases. Some reports demonstrated that including lumbar puncture and spinal CSF leak may cause SDH. Low intracranial pressure and excessive CSF outflow are the key factors. In addition, it is well known that spontaneous intracranial hypotension is sometimes associated with chronic subdural hematoma (16–18). Therefore, we speculated that this distal supratentorial SDH could be the cause of an excessive and abrupt release of CSF. During surgery, a non-standard position of the head and improper manipulation of aspirators could also produce an abrupt and excessive release of CSF. Moreover, a tortuous VBA usually occupies a major surgical field. To achieve an appropriate operating space, more CSF should be released during surgery. In 2 patients, after the complete release of CSF, a stream of bloody CSF oozed out again at the same time. We suggested releasing CSF as slowly as possible and keeping the head in a standard position during surgery. Furthermore, we observed cerebral atrophy through preoperative CTs in 2 patients who developed distal supratentorial SDH (Figure 6). Some reports demonstrated the association between cerebral atrophy and SDH (19–21), and the discharge of CSF can lead to brain sagging, which can tear congested bridging veins, hence enabling the formation of SDH (18, 22). These two factors together posed a high risk of SDH. Of 6 distal SDH patients, 1 experienced consciousness disturbance, and a CT scan demonstrated extensive frontotemporal SDH: the patient immediately underwent hematoma evacuation and drainage. The other 5 patients recovered with conservative therapy. Most patients with SDH can have a favorable prognosis thanks to it, but a few of them need surgery because of consciousness disturbance and hemorrhaging in a large area.

Postoperative ICH in MVD has a poor prognosis. Hypertension is the most important risk factor for the development of ICH and more than doubles the risk of ICH (23–27). Therefore, hypertension is an important basis for postoperative hemorrhage. In addition, another study suggested that blood pressure variability was associated with the clinical outcome of ICH patients (28). In our study, there was no significant correlation between the Hemphill Score and the SD, CV range of SBP, which responded to the fluctuation of blood pressure, however we still considered that blood pressure variability was associated with ICH. A five-minute interval between two measurements of blood pressure during surgery was too long because manipulations in MVD can affect blood pressure in an extremely short time. Especially when we explored the roots of the trigeminal nerve, an oculocardiac reflex may suddenly cause a decrease in blood pressure and heart rate. This type of change can only be recorded by continuous arterial blood pressure. Additionally, trachea extubation after anesthesia may cause remarkable fluctuations in blood pressure. Further studies are needed to confirm the exact correlation between blood pressure variability and postoperative hemorrhage after MVD using the biomedical glue sling technique. Also, vertebrobasilar tortuosity has been a risk factor for ICH. Passero et al. studied 156 patients with VBA tortuosity and found that 28 of them developed ICH. Among these patients, there was also a significant proportion of long-term use of oral antiplatelet drugs, which further increased the risk of bleeding (29). We inferred that there were some changes in the hemodynamics of patients with tortuous VBA in our study, but concrete mechanisms involving postoperative hemorrhage.

## Conclusion

The incidence of postoperative hemorrhage after MVD using the biomedical glue sling technique is marginally higher than traditional MVD and most cases have a favorable prognosis. Postoperative symptoms are the main area of concern, and changes in symptoms usually suggest the emergence of a hemorrhage. Several factors, including surgical procedures, the release of CSF, and blood pressure might be associated with hemorrhages. The biomedical glue sling technique is an efficient approach to treating complicated cranial nerve diseases, and more examples are needed to demonstrate the safety of this technique.

## Data Availability

The raw data supporting the conclusions of this article will be made available by the authors, without undue reservation.
